# Magnetic Fe_3_O_4_-Based Sandwich-Type Biosensor Using Modified Gold Nanoparticles as Colorimetric Probes for the Detection of Dopamine

**DOI:** 10.3390/ma6125690

**Published:** 2013-12-05

**Authors:** Zhiyong Wang, Yanyan Bai, Wenchao Wei, Ning Xia, Yuhui Du

**Affiliations:** College of Chemistry and Chemical Engineering, Anyang Normal University, Anyang 455000, Henan, China; E-Mails: ybhxx1@163.com (Y.B.); wwchxx@sina.com (W.W.); yhduhxx@163.com (Y.D.)

**Keywords:** gold nanoparticles, magnetic particle, biosensor, dopamine, colorimetric assay

## Abstract

In this work, we designed a visual biosensor for dopamine (DA) detection using magnetic Fe_3_O_4_ particles and dithiobis(sulfosuccinimidylpropionate)-modified gold nanoparticles (DTSSP-AuNPs) as the recognition elements. Specifically, DA molecules were assembled onto the surface of DTSSP-AuNPs via the amine coupling reaction between the amino group of DA and activated carboxyl group of DTSSP. Accordingly, DA-anchored DTSSP-AuNPs were captured by Fe_3_O_4_ through the interaction of catechol and iron. In a magnetic field, the formed Fe_3_O_4_-DA-DTSSP-AuNPs conjugates were easily removed from the solution, leading to fading of the AuNPs suspension and decrease of the UV/Vis signal. As a result, a detection limit of 10 nM for DA was achieved. The theoretical simplicity and high selectivity demonstrated that the sandwich-type strategy based on Fe_3_O_4_ and AuNPs would lead to many colorimetric detection applications in clinical study by rationally designing the surface chemistry of AuNPs and Fe_3_O_4_.

## 1. Introduction

Gold nanoparticles (AuNPs) based colorimetric assays have been widely applied in a variety of research fields due to the high extinction coefficient (3–5 orders of magnitude higher than those of organic dye molecules [1]) and the unique size-dependent optical property of AuNPs [2,3,4,5,6,7]. Such methods are very promising in that they require very simple sample handling procedures and minimum instrumental investment and can be conducted in the field with portable devices. The rational design of the surface chemistry of AuNPs can promote specific interactions between the receptors and analytes and, as a result, renders the measurements highly selective [8]. Magnetic particles (MPs) are also one of the most popular materials that have been widely exploited in medical diagnostics, controlled drug release and separation technologies [9,10]. Even before the term “nanotechnology” was popular, iron oxide (Fe_3_O_4_) nanoparticles were used to magnetically isolate and purify proteins, DNA, viruses and even whole mammalian cells [11]. Recently, bifunctional Au-Fe_3_O_4_ nanoparticles, recognized as a major advancement in nanobiotechnology generating, from the two components, excellent surface chemistry, special optical properties, and superparamagnetic properties, have been synthesized by various strategies [12,13,14,15,16,17,18], and many applications in a wide variety of fields have been found, such as DNA hybridization assay and protein detection [19,20,21,22,23].

Dopamine (DA) is a monoamine neurotransmitter distributed in the central neural system brain tissues and body fluids of mammals. It plays pivotal roles in the function of the central nervous, renal, hormonal and cardiovascular system. Detection of DA is important in diagnoses, monitoring, prevention and treatment of some certain diseases, such as Parkinson’s disease, Alzheimer’s disease, Huntington’s disease, epilepsy, pheochromocytoma and neuroblastoma [24]. Herein, we reported a sandwich-type strategy for DA detection using magnetic Fe_3_O_4_ particles and dithiobis(sulfosuccinimidylpropionate) (DTSSP)-modified AuNPs (DTSSP-AuNPs). This method is based on the following two facts: (1) DA can form stable, robust anchor onto the surface of iron oxide by the interaction of catechol and iron, facilitating the conjugation of biomolecules onto the Fe_3_O_4_ surface via the amine coupling reaction between DA and biomolecules [25,26,27,28,29,30]; and (2) the amine-reactive succinimidyl residues on the surface of AuNPs can react selectively with the primary amine group of DA [4,31]. As a result, decrease in the UV/Vis absorbance of DTSSP-AuNPs suspension was observed in the external magnetic field when DA and Fe_3_O_4_ were successively added to the DTSSP-AuNPs suspension. The method is sensitive and selective to DA detection without the requirement of expensive and complicated instruments. To the best of our knowledge, this is the first magnetic Fe_3_O_4_-based biosensor with AuNPs as colorimetric probes.

## 2. Experimental Section

### 2.1. Reagents and Materials

Dithiobis(sulfosuccinimidylpropionate) (DTSSP) was purchased from Chemsky International Co., Ltd. (Shanghai, China), dihydroxyphenylacetic acid (DOPAC), glutamic acid, tyrosine and trisodium citrate were obtained from Sigma-Aldrich (Shanghai, China). Norepinephrine and dopamine hydrochloride were obtained from Aladdin reagent Inc. (Shanghai, China). All of other reagents were analytical-grade reagents and were used without further purification. DA and norepinephrine were prepared with HCl solution (pH 2) and diluted to the desired concentration with phosphate buffer solution (PBS, 2 mM, pH 7.2) containing 0.1 mM Na_2_SO_3_. The other small-molecule organic chemicals were dissolved in deionized water. Unless otherwise noted, the reactions were conducted at room temperature.

### 2.2. Synthesis of DTSSP-AuNPs

The citrate-stabilized AuNPs were prepared using a trisodium citrate reduction method. The DTSSP-modified AuNPs were prepared by ligand-exchange reaction between DTSSP and citrate-stabilized AuNPs, as in previous reports [4,31,32]. Briefly, trisodium citrate (5 mL, 38.8 mM) was rapidly added to a boiling solution of HAuCl_4_ (50 mL, 1 mM), and the solution was boiled continually for an additional 30 min to yield a wine-red solution. Ligand-exchange reaction was performed at room temperature by mixing 10 mL of the diluted AuNPs suspension with 10 µL of DTSSP solution under stirring for 30 min. After filtering the solution through a 0.45-μm membrane filter to remove the precipitate, the filtrate was used for DA assay. The particle concentration of the AuNPs solution was calculated by using a molar absorptivity of 2.7 × 10^8^ M^−^^1^·cm^−^^1^ at 520 nm [33]. The optimal molar ratio of DTSSP to AuNPs was presented in the Result section. The morphology of DTSSP-AuNPs was characterized by an FEI Tecnai G2 T20 transmission electron microscope (TEM).

### 2.3. Synthesis of Magnetic Fe_3_O_4_ Particles

Magnetic Fe_3_O_4_ particles were prepared via the co-precipitation [34]. Briefly, FeCl_3_·6H_2_O (5.40 g) and FeSO_4_·7H_2_O (2.78 g) were dissolved under a N_2_ atmosphere in 100 mL of deoxygenated water, which had been bubbled for 15 min with nitrogen gas. After the Fe^3+^/Fe^2+^ mixed solution was stirred in a water bath at 80 °C for 30 min, aqueous ammonia (28%) was dropped into the solution at 80 °C with stirring until pH 9 was reached. The mixture was then stirred continuously for 30 min in the water bath at 80 °C. With the completion of the reaction, an external magnet was used to separate the Fe_3_O_4_ from the sample solution. The isolated Fe_3_O_4_ MPs were washed repeatedly with hot water to remove untreated impurities and then dried in an oven at 90 °C for 30 min.

### 2.4. Detection of DA

Three hundred and thirty microliters of dispersions of the DTSSP-AuNPs were first diluted with 660 µL PBS. Then, DA (10 µL) was added to the diluted solution to react with DTSSP-AuNPs for 30 min. This step is followed by the addition of 1 mg of magnetic Fe_3_O_4_ particles. Color change was observed with the naked eyes and the absorption spectra were recorded with a Cary 50 UV/Vis spectrometer. The photographs were taken with a Sony Cyber-shot digital camera. Reaction and detection were conducted at room temperature.

## 3. Results and Discussion

### 3.1. Mechanism of DA Detection

**Figure 1:**
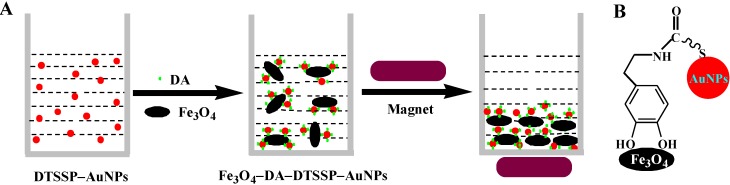
Schematic illustration of the strategy for dopamine (DA) detection using dithiobis(sulfosuccinimidylpropionate)-modified gold nanoparticles (DTSSP-AuNPs) and Fe_3_O_4_ magnetic particles (MPs) (**A**) and the interaction between DA, Fe_3_O_4_ and DTSSP-AuNPs (**B**).

In view of the magnetic nature of Fe_3_O_4_, Fe_3_O_4_-based biosensors have been developed to detect DNA and protein with carbon nanotubes and the quantum dot as the recognition elements [34,35]. The principle of our strategy with colorimetric AuNPs as the recognition elements is shown in Figure 1. The Fe_3_O_4_ MPs and DTSSP-AuNPs can form a sandwich structure of Fe_3_O_4_-DA-DTSSP-AuNPs. DA molecules were first assembled onto the surface of DTSSP-AuNPs via the standard amine coupling reaction between the amino group of DA and activated carboxyl group of DTSSP. Accordingly, DA-anchored DTSSP-AuNPs can be separated by Fe_3_O_4_ MPs in the external magnetic field through the interaction of catechol and iron [25,26,27,28,29,30], leading to fading in the color of the DTSSP-AuNPs suspension and a decrease in the UV/Vis absorbance. The method based on this concept should be very sensitive because Fe_3_O_4_ MPs have excellent separation ability and AuNPs have high extinction coefficient.

### 3.2. Colorimetric Assay for DA

**Figure 2:**
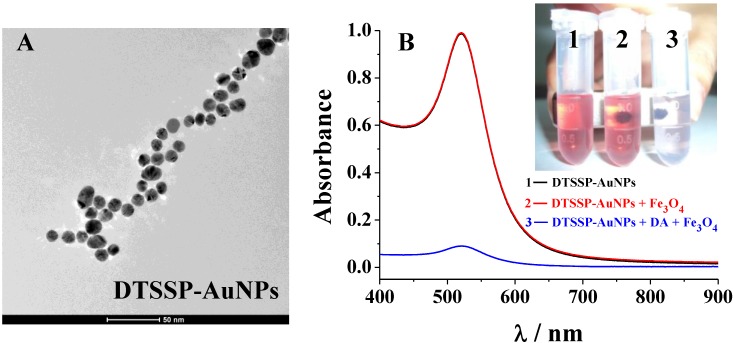
(**A**) Transmission electron microscope (TEM) of DTSSP-AuNPs and (**B**) UV/Vis absorption spectra of DTSSP-AuNPs with and without addition of DA (4 µM) and Fe_3_O_4_ MPs. The inset in panel B shows the visual color change.

The synthesized DTSSP-AuNPs were first characterized by UV/Vis spectroscopy and TEM. The average size of the DTSSP-AuNPs was determined to be 12.8 nm from the TEM image (Figure 2A). The red DTSSP-AuNPs suspension exhibited an absorption peak at 520 nm (A_520_), which was ascribed to its surface plasmon resonance (vial 1 and black curve in Figure 2B). No obvious change was observed upon the addition of Fe_3_O_4_ (vial 2 and black curve), indicating that AuNPs did not absorb onto the surface of Fe_3_O_4_ MPs. However, when DA and Fe_3_O_4_ were added successively to the solution of DTSSP-AuNPs in the external magnetic field, the red color disappeared (vial 3). Note that DA did not cause the aggregation and color change of DTSSP-AuNPs in the absence of Fe_3_O_4_. Meanwhile, the DA-induced conjugate of DTSSP-AuNPs and Fe_3_O_4_ was also monitored by UV/Vis spectroscopy. As shown in the blue curve, the original absorbance of AuNPs at 520 nm almost dropped to the background level with the addition of DA and Fe_3_O_4_.

**Figure 3:**
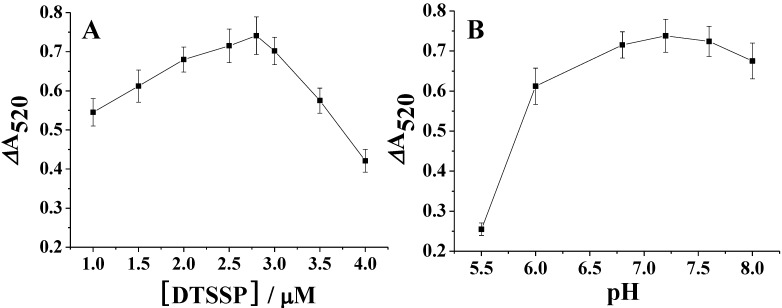
(**A**) Signal intensity variation of AuNPs functionalized by different amounts of DTSSP toward the addition of DA (2 µM) and Fe_3_O_4_ MPs; (**B**) Effect of pH on the ΔA_52__0_.

We also optimized the molar ratio of DTSSP to AuNPs by measuring the absorbance change of DTSSP-AuNPs in the presence of DA and Fe_3_O_4_. ΔA_520_ (ΔA_520_ = A_520_′ − A_520_^0^, where A_520_′ represents the absorbance of AuNPs with modification of different amounts of DTSSP in the presence of DA and Fe_3_O_4_, and A_520_^0^ represents that in the absence of DA) was used here to evaluate the performances of the sensor. As shown in Figure 3A, ΔA_520_ reaches maximum at the DTSSP concentration of around 2.8 µM. This is understandable since free DTSSP in the solution could compete with DTSSP-AuNPs to react with DA, leading to the decrease in the amount of DA molecules anchored onto AuNPs surface. From the result, DTSSP at the concentration of 2.8 µM could be completely absorbed by 3.6 nM AuNPs. Thus, the average number of DTSSP molecules per gold nanoparticle was calculated to be around 778. Moreover, solution pH affects not only the stability of AuNPs and DTSSP, but also the reactivity of DA to DTSSP. The effect of pH on the ΔA_520_ was also examined over a range from 5.5 to 8.0. As shown in Figure 3B, ΔA_520_ reaches a maximum at pH around 7.2. Thus, we chose pH 7.2 PBS solution as the reaction medium. The signal decreases remarkably at low and high pH, probably due to the poor reactivity of amine to DTSSP and the ionization of DTSSP molecules [36].

### 3.3. Sensitivity

**Figure 4:**
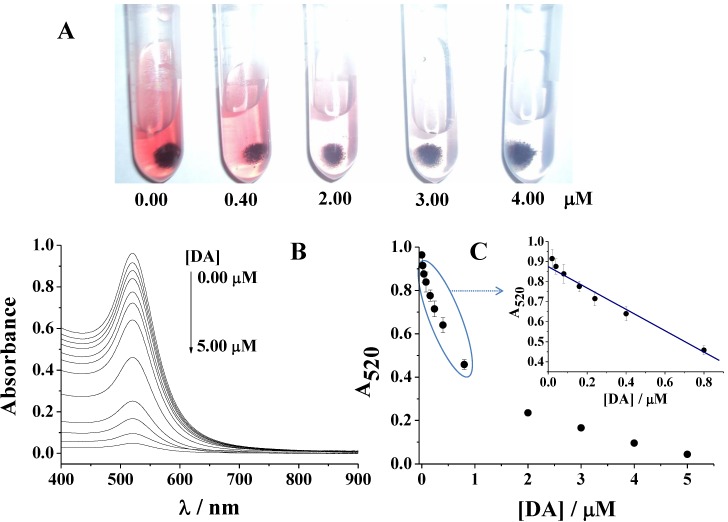
(**A**) Color change with increasing DA concentrations; (**B**) Absorbance response for different concentrations of DA; (**C**) The corresponding plot of A_520 _*vs.* DA concentration from 0, 0.02, 0.04, 0.08, 0.16, 0.24, 0.40, 0.80, 2.00, 3.00, 4.00 to 5.00 µM.

To demonstrate the performance of the sensor for naked eye detection of DA by the mechanism mentioned above, different amounts of DA were added to the DTSSP-AuNPs solution. The results were presented in Figure 4A. Upon the addition of increasing concentrations of DA, the red color of DTSSP-AuNPs faded gradually. These results were also confirmed by the UV/Vis spectroscopy. As shown in Figure 4B, with the addition of increasing concentrations of DA to the solution of DTSSP-AuNPs, obvious decrease in the absorption peak at 520 nm was observed. A linear relationship was found between A_520_ and DA concentration over the range of 0.02–0.80 µM, which can be expressed using A_520_ = 0.87 − 0.53 [DA] (µM) (R^2^ = 0.99). The detection limit for DA was estimated to be 10 nM. 

### 3.4. Selectivity

**Figure 5:**
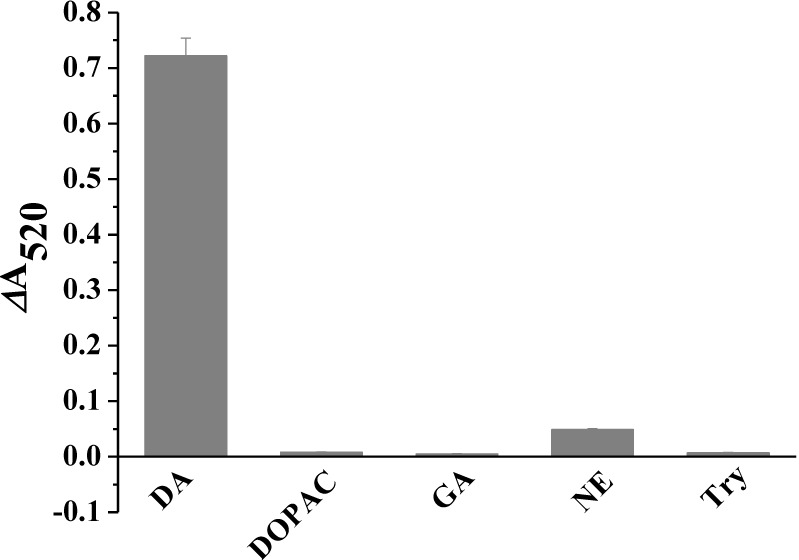
Selectivity of the sensing protocol. DOPAC: dihydroxyphenylacetic acid; GA: glutamic acid; NE: norepinephrine; Tyr: tyrosine. The concentrations of DA and interferences are 2 and 20 µM, respectively.

The selectivity of the present approach was evaluated by testing other members of the catechol family and amino acids that may coexist in the body fluids of mammals, such as DOPAC and norepinephrine, glutamic acid and tyrosine. Interestingly, we found that no obvious intensity changes were observed when all of the above four compounds were added to the dispersion of AuNPs (Figure 5). For glutamic acid and tyrosine, this result is understandable because they have no (or poor) affinity to Fe_3_O_4_ although they can absorb onto the surface of DTSSP-AuNPs through the formation of amino bond. For DOPAC, a reasonable explanation is that it has no amine group and cannot be assembled onto the DTSSP-AuNPs surface via the amine coupling reaction, although it can be captured by Fe_3_O_4_. However, although NE can also be captured by Fe_3_O_4_ and has primary amine group, its addition induced a slight change in the intensity. We presume that this lack of obvious change is probably due to the differential reactivity of the amines with DTSSP on the surface of AuNPs [4,24].

## 4. Conclusions/Outlook

In this study, we report for the first time, a sandwich-type biosensor using magnetic Fe_3_O_4_ and colorimetric AuNPs. In the work, DTSSP-AuNPs were connected with Fe_3_O_4_ MPs by DA and formed a sandwich structure of Fe_3_O_4_-DA-DTSSP-AuNPs. The method was developed to detect DA by observing the color change of DTSSP-AuNPs suspension and by measuring the UV/Vis signal. A detection limit of 10 nM for DA was achieved. The theoretical simplicity and high selectivity reported herein demonstrated that the detection strategy will likely find many applications in clinical study by rationally designing the surface chemistry of AuNPs and Fe_3_O_4_.
